# Unveiling the switching mechanism of robust tetrazine-based memristive nociceptors *via* a spectroelectrochemical approach†

**DOI:** 10.1039/d5sc02710a

**Published:** 2025-06-03

**Authors:** JiYu Zhao, Kun Liu, Wei Zeng, Zhuo Chen, Yifan Zheng, Zherui Zhao, Wen-Min Zhong, Su-Ting Han, Guanglong Ding, Ye Zhou, Xiaojun Peng

**Affiliations:** a College of Materials Science and Engineering, Shenzhen University Shenzhen 518060 P. R. China; b Institute for Advanced Study, Shenzhen University Shenzhen 518060 P. R. China yezhou@szu.edu.cn; c State Key Laboratory of Fine Chemicals, Frontiers Science Center for Smart Materials, Dalian University of Technology Dalian 116024 P. R. China pengxj@dlut.edu.cn; d Department of Applied Biology and Chemical Technology, Research Institute for Smart Energy, The Hong Kong Polytechnic University Hung Hom Hong Kong SAR 999077 P. R. China; e State Key Laboratory of Radio Frequency Heterogeneous Integration, Shenzhen University Shenzhen 518060 P. R. China dinggl@szu.edu.cn

## Abstract

Threshold-switching memristors exhibit significant potential for developing artificial nociceptors as their working principles and electrical characteristics closely mimic biological nociceptors. However, the development of high-performance artificial nociceptors is hindered by the randomness of conductive filament (CF) formation/rupture, caused by low-quality resistive switching (RS) films, and complex and uncontrollable RS mechanisms. Organic small-molecule materials are favored in electronic devices for their designability, low cost, easy synthesis, and high stability. In this study, we meticulously designed two D–π–A–π–D structured molecules, designated as TZ-1 and TZ-2, to serve as the RS layer in artificial nociceptors. By precisely modulating the electron-donating ability of the donor groups in these molecules, some key electrical properties of the memristor, such as the low SET voltage (0.42 V) and variation (0.055), high current ON/OFF ratio (∼10^−6^) and nanosecond level switching time (60 ns), can be successfully optimized. Moreover, a spectroelectrochemical strategy was employed for the first time to investigate the RS mechanism at the molecular level, elucidating the critical role of molecular design in modulating the device's working principles and electrical characteristics. The optimized memristor is capable of accurately emulating the four key behaviors of nociceptors. This achievement not only advances the application of organic materials in neuromorphic devices but also opens up new possibilities for the specialized customization of nociceptors.

## Abstract 2

Two organic small molecules were designed and synthesized to serve as memristive artificial nociceptor in memristors.

## Graphical abstract

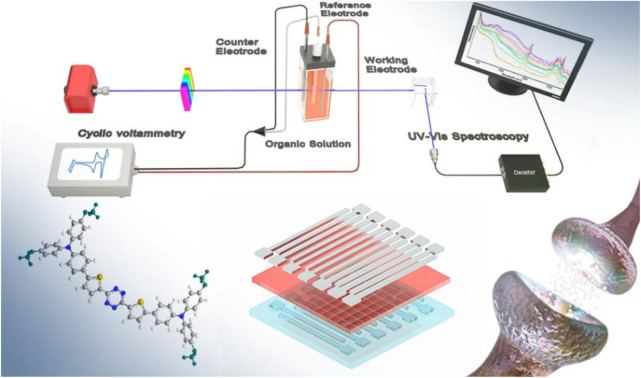

## Introduction

The sensory nervous systems serve as a vital conduit for communication between the human body and its external environment, enabling the perception of necessary conditions for biological survival.^1–4^ Specifically, in a sensory nervous system, sensory receptors receive external stimuli and transmit signals to neurons for analysis and processing.^3^ The nociceptor is a specific type of sensory receptor in the sensory nervous system.^4–6^ It targets the sensing and perception of harmful stimuli for avoiding danger, reflected in four typical special features, that is, “threshold”, “no adaptation”, “relaxation”, and “sensitization”.^5–11^ Owing to the easily achievable threshold switching (TS) behavior, memristors with an electrochemical metallization (ECM) mechanism are promising for constructing artificial nociceptor systems.^6,8,12^ In this system, when the input electrical signal, which can be converted through an in-/near-sensor strategy from an external noxious stimulus, surpasses the operational threshold of the memristor, the artificial nociceptor will generate a warning signal. This biomimetic threshold response mode effectively filters out numerous harmless external stimuli, facilitating high-efficiency and low-power data processing while holding significant implications for intelligent robot construction.

Up to now, a large variety of materials have been utilized as resistive switching (RS) layers in memristors, including perovskites,^7,13^ metal oxides,^3,6,14^ and organic materials.^15–17^ We have compiled a selection of relevant literature and compared four key parameters and nociceptive thresholds (Table 1). Through a systematic comparative study of the key characteristics of nociceptors, it was found that organic, inorganic and hybrid systems exhibit similar performance advantages. However, leveraging the designable structural characteristics of organic materials, organic memristors achieve a notably high ON/OFF ratio while significantly reducing the turn-on voltage threshold, a feature that effectively highlights their unique advantages in the field of biomimetic neural simulation. For example, Wang *et al.* constructed a molecular-scale reconfigurable device through molecular design, achieving electronic functions such as variable resistors and diodes.^18^ Through precise molecular-level regulation, organic devices also demonstrate substantial development potential in the fields of neuromorphic computing^19,20^ and reservoir computing.^21^ Although the ECM-based memristor has excellent performance in mimicking the behaviors of a biological nociceptor, factors such as the uniformity of the thin film, the random formation/fracture of conductive filaments (CFs), and others can affect the accuracy and reliability of the simulated nociceptor.^14,22–24^ Among them, organic materials receive much attention due to their excellent intrinsic flexibility, solution-treatable feature, and structural designability. However, traditional investigation models (such as morphological characterization and first-principles calculations) often fail to consider the functional groups of organic materials, thus falling short in adequately addressing the underlying mechanisms.^22^ There is an urgent need to conduct mechanism studies on organic materials. Spectroelectrochemical technology, which integrates spectroscopy with electrochemical analysis, provides valuable insights into reaction mechanisms, intermediates, and associated details by synchronously measuring electrochemical and spectroscopic signals. Therefore, this technology can be applied for elucidating the influence of molecular properties on ECM mechanisms.

**Table 1:** Organic, inorganic and hybrid material nociceptors using memristors

Device structure	*I*_HRS_ (*V*_Read_)	*I*_LRS_	*I*_ON/OFF_	*V*_SET_	Nociceptive threshold	Ref.
Ag/ZrO_*x*_/Pt	—	10 μA (*I*_cc_)	10^5^	≈0.4 V	6 V	36
Ag/2D-GaO_*x*_/SiO_*x*_/Si	—	—	10^4^	≈2.3 V	5 V	37
P-Si/CZO/Au	≈10 nA (3 V)	≈10 μA	10^3^	≈15 V	11 V	38
Ag/CιC/ITO	<10 nA (0.2 V)	100 μA	>10^4^	≈0.4 V	0.6 V	39
Ag/PMMA & CsPbCl_3_/ITO	≈100 nA (0.1 V)	5 mA	10^3^	0.85 V	0.9 V	40
ITO/ZnO/TiO_*x*_/ITO	≈10 nA (0.1 V)	1 mA	10^4^	1.23 V	0.6 V	41
Ag/PMMA&Cs_3_Bi_2_I_9_/ITO	<100 nA (0.1 V)	1 mA (*I*_cc_)	10^3^	≈0.6 V	0.6 V	42
Pd/MAPbI_3_/ITO	<10 nA (—)	100 μA (*I*_cc_)	10^4^	≈1.5 V	1.5 V	43
Pt/LiSiO_*x*_/TiN	<500 nA (0.3 V)	5 mA	10^4^	≈1 V	1 V	44
Al/2DPTPAK^+^TAPB/Ag	≈31.8 nA (0.1 V)	≈721 μA	2 × 10^4^	≈1.74 V	—	45
Ag/TZ-1/ITO	<1 nA (0.05 V)	≈10 μA	>10^4^	0.39 V	0.6 V	This work

Organic molecules exhibit a relatively wide range of applications in the electronic device field, including transistors,^25,26^ solar cells,^27,28^ sensors,^29,30^ and memristors.^31–34^ For example, in the field of memristors, Yi *et al.* fabricated an organic polymeric memristor using poly[2-methoxy-5-(3′,7′-dimethyloctyloxy)-1,4-phenylenevinylene] (MDMO-PPV) as an RS material for mimicking biological synaptic properties (including potentiation/depression, spike-rate-dependent plasticity, spike-timing-dependent plasticity).^32^ Furthermore, the straightforward configuration of small molecules and the effortless fabrication of high-quality films (simple vacuum evaporation or solution spin-coating processes)^35^ make it more conducive to the verification of mechanistic models. In this study, modified spectroelectrochemical technology was employed for in-depth analysis of the effects of the small-molecule functional groups on the ECM mechanism, demonstrating that this approach is an effective method for elucidating the device RS mechanism. Leveraging the multiple advantages (*i.e.* simple synthesis route and high-quality films), and combining the structure/property designability, two organic tetrazine (TZ) molecules with different electron-donating groups were designed and synthesized to controllably regulate the electron transmission properties. These novel TZ molecules can serve as an RS layer for fabricating ECM-based memristors, which are also applicable to the exploration of underlying mechanisms. Additionally, memristors incorporating these TZ molecules can emulate key properties of biological nociceptors, including “threshold”, “no adaptation”, “relaxation”, and “sensitization”.

## Results and discussion

### Molecular design and structural analysis

The TS behaviors can be achieved and modulated by adjusting the electron-donating ability of the substituent groups of the small molecules in the RS layer. Generally, in the molecular design of semiconductor materials, lowering the energy levels of frontier molecular orbitals (FMOs) and extending the π-conjugated structures are effective strategies for improving charge transport properties.^35,46^

**Fig. 1:**
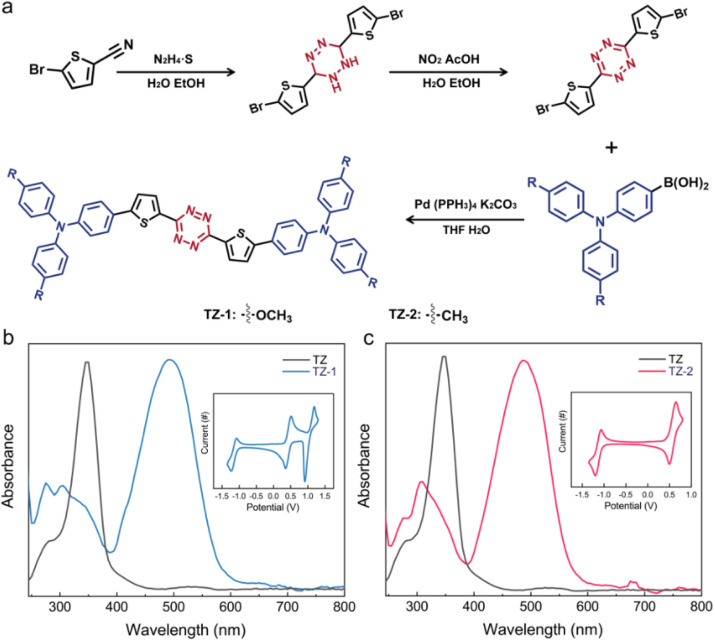
Molecule synthesis pathway and spectral expression. (a) Synthesis routes for TZ-1 and TZ-2. (b and c) UV-vis absorption spectra of TZ, TZ-1 (b) and TZ-2 (c). Dissolved in dichloromethane (DCM); wavelength: 800–240 nm. Insets: CV curves of TZ-1 (b) and TZ-2 (c); DCM containing 0.1 M Bu_4_N[PF_6_] as the supporting electrolyte, Ag/AgNO_3_ as the reference electrode; ferrocene (Fc/Fc^+^) was used as an internal reference. Scan rate: 50 mV s^−1^.

Tetrazine compounds exhibit high electron affinity and demonstrate excellent stability under electrochemical conditions, rendering them ideal as electron transport materials that can boost electron injection and transfer efficiency.^47–51^ As a bridge functional group, thiophene has been demonstrated to have excellent electron transport properties, connecting electron donors and acceptors, and facilitating charge transfer between molecules.^52^ In this work, we explored different electron-donating groups, namely a methyl group with triphenylamine and a methoxy group with triphenylamine.^53^ The synthesis procedure is illustrated in Fig. 1a. All molecular structures of the new compounds were characterized and confirmed by ^1^H NMR and mass spectroscopy (ESI,† Materials and synthesis). The thermogravimetric analysis (TGA) results for both molecules in Fig. S1† indicate that these small molecules maintain their structural integrity even at high temperatures (200 °C), demonstrating their robust and dependable environmental stability.

To gain further insights into the photophysical characteristics, UV-vis (ultraviolet-visible absorption spectroscopy) and CV (electrochemical cyclic voltammetry) were employed. As illustrated in Fig. 1b and c, compared to that of TZ (maximum absorption wavelength: 347 nm), the UV-vis spectra for TZ-1 and TZ-2 have two obvious absorption peaks (TZ-1: 305 and 498 nm; TZ-2: 310 and 487 nm), of which the peaks at 305 nm and 310 nm can be attributed to the absorption blue shift of the π–π* transition of TZ (300 nm), and those at 498 and 487 nm to intramolecular charge transfer.^47,49^ The maximum absorption wavelength of TZ-1 exhibits a red shift of approximately 11 nm relative to TZ-2, suggesting that the strong electron-donating group effectively stabilizes the positive charges within the molecule and enhances charge transfer.^53,54^ From the CV results depicted in the inset figures in Fig. 1b and c, the reversible oxidation peaks of TZ-1 and TZ-2 are observable at 0.51 and 0.58 eV, respectively, while their reversible reduction peaks are located at 0.35 and 0.49 eV, respectively. The fact that TZ-1 exhibits a lower oxidation potential compared to TZ-2 indicates its greater propensity to donate electrons, thereby facilitating charge transfer. The HOMO, LUMO and energy band gaps can be derived from the UV-vis and CV results (ESI, Energy band gaps of TZ molecules) (Fig. S2†).

Experimental outcomes confirm the successful synthesis in this study of two organic small molecules possessing identical LUMO energy levels *via* a meticulous molecular design approach, effectively eliminating the disruption caused by energy level barriers to electron transport. This achievement furnishes an ideal sample for advancing spectroelectrochemical research. Not only does this design highlight the potential of spectroelectrochemical techniques in probing memristor mechanisms but also establishes a robust platform for future investigations into the interplay between electron transport behavior and molecular structure.

### Memristor fabrication and electrical characteristics

**Fig. 2:**
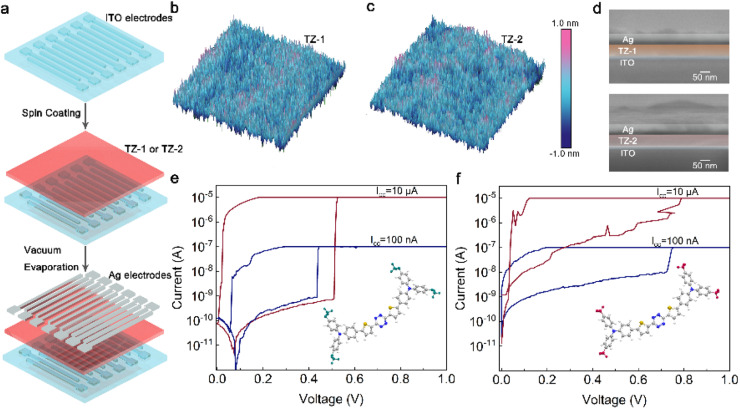
Device preparation, thin film and electrical characterization. (a) Schematic illustration of the fabrication process for the TZ-based TSM using an all-solution approach and thermal evaporation. (b and c) AFM images of TZ-1 (b) and TZ-2 (c) films. (d) Cross-section SEM images of the TZ-1 (top) and TZ-2 (bottom) based TSMs. RS behaviors of the TZ-1/2 TSMs under different *I*_cc_ values (100 nA and 10 μA): (e) TZ-1; (f) TZ-2; the ball-and-stick models of TZ-1 and TZ-2 are shown in the insets.

Volatile memristors exhibiting TS behavior are well-suited for developing artificial nociceptors and emulating the functionalities of biological nociceptors. To verify the electrical behaviors of the synthesized novel molecules, and develop stable and reliable artificial nociceptors, TS memristors (TSMs) with a cross-bar sandwich structure were fabricated, and their electrical properties were systematically investigated. The device structure consists of pre-patterned indium tin oxide (ITO, 23 nm) as the bottom electrodes (BEs) and silver (Ag, 50 nm) as the top electrodes (TEs, Fig. 2a). The synthesized TZ molecules were employed as RS materials, and the two molecules were utilized as independent RS layers, which were fabricated *via* spin-coating acetonitrile solutions of these molecules using an all-solution approach (ESI,† Device fabrication and characterization). According to the atomic force microscopy (AFM) results in Fig. 2b and c, the TZ molecule films exhibit uniform and flat properties with sub-nanometer roughness [(root-mean-square roughness (*R*_a_), TZ-1: 0.429 nm; TZ-2: 0.629 nm)]. The device structures were characterized by cross-section scanning electron microscopy (SEM). According to the results in Fig. 2d, the thickness of the TZ-1/2 molecule films is approximately 60 nm, which is also verified by the AFM measurements (Fig. S3†).

Fig. 2e and f display the typical RS behaviors of TZ-1 and TZ-2, respectively. In contrast, the HRS of the TZ-2-based TSM increased with increasing *I*_cc_, indicating relatively weaker stability. Additionally, the TZ-1 TSM demonstrated a lower *V*_SET_ than that of the TZ-2 TSM, which facilitates low-energy operation. Consequently, TZ-1 enables the formation of stronger and more stable CFs, offering greater versatility for biomimetic simulations.^55,56^

Based on the conclusions from UV-vis spectra and CV, and incorporating the device's electrical properties, it becomes evident that the highly potent electron-donating group of TZ-1 and the easy charge transfer property facilitate the realization of a reduced *V*_SET_, a notably stable HRS, and a highly tunable *I*_ON/OFF_. Therefore, the strategy of structurally designing organic small molecules to modulate device electrical performance in this study can establish a new paradigm for advancing organic electronic materials.

### Device working mechanism analysis

The FMOs of the synthesized TZ molecules were obtained from DFT calculations (ESI, Study of device working mechanisms, Note S1†).^53,57–59^ According to the FMO ESP (electrostatic potential) results in Fig. S4,† the thiophene, as a bridge functional group consisting of a conjugated skeleton with a continuous cloud of electrons, constructed a free pathway for charge transfer. Compared to TZ-2, TZ-1 exhibits greater energy depth due to the introduction of a methoxy group with triphenylamine as the electron donor. This modification facilitates more efficient charge transfer and enhances charge transport capacity.

**Fig. 3:**
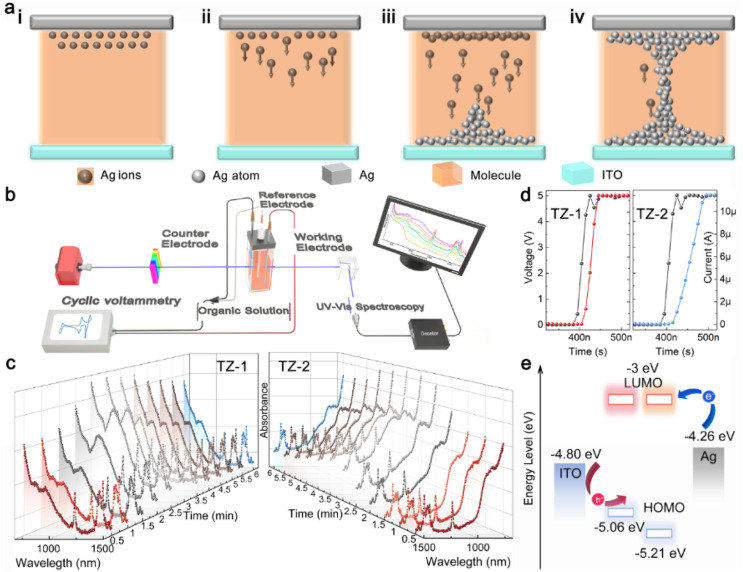
Migration mechanism of Ag^+^ and spectroelectrochemistry. (a) The schematic diagram of the ECM RS mechanism [oxidation (i), migration (ii), reduction (iii), and formation of CFs (iv)] of the TZ-based TSMs. (b) The spectroelectrochemical setup (UV-vis spectroscopy coupled to CV) used to investigate the reaction mechanism between the active electrode atoms (Ag) and the organic small molecules. (c) *In situ* UV-vis absorption spectra of TZ-1/2 in the applied bias cycles (left: TZ-1; right: TZ-2). UV-vis absorption spectra from 1500 to 600 nm; CV curves at a scan rate of 7 mV s^−1^, from 0 to 1.2 V cycles. (d) Switching time for TZ-1 and TZ-2. TZ-1: 60 ns; TZ-2: 110 ns; amplitude: 5 V; width: 500 ns: interval: 10 ns. (e) Energy levels for TZ-1 (left), TZ-2 (right), Ag, and ITO.

Based on the ECM mechanism and incorporating a molecular structure design strategy, the device working mechanisms of these two TZ-based TSMs were investigated using spectroelectrochemical techniques to elucidate the differing electrical performances of the two similar molecules at the molecular level. The ECM RS mechanism commonly involves the redox process of Ag atoms (ESI, Study of device working mechanism, Note S2†), containing three steps: oxidation reaction of Ag atoms to Ag^+^ (Fig. 3a(i)), migration under the voltage bias (Fig. 3a(ii)), and the reduction of Ag^+^ to Ag atoms for forming metallic CFs (Fig. 3a(iii) and (iv)).^60–63^

Spectroscopic techniques, including Raman and X-ray diffraction (XRD) analysis (ESI, Fig. S5c, d, and S6†), were initially employed to investigate the structure, composition, and molecular state of the TZ films. Raman spectra showed less than 5% variation (Fig. S7†) in all characteristic peaks over the full spectral range. Based on these findings, we believe that the TZ molecule in DCM solution can be utilized to investigate the device RS mechanism, even though they are not in a solid film state (ESI, Study of device working mechanisms, Note S3†).

Spectroelectrochemical technology examines molecular structures and electron transfer during electrochemical reactions, due to its capacity to monitor real-time variations in reactant species during reactions, capture detailed molecular vibration spectra at the electrode/solution interface, and offer profound insights into material properties at the molecular level. UV-vis spectroelectrochemical studies, by tracking alterations in UV-vis light absorption during the reaction, is well-suited for studying redox reactions. This technique closely corresponds to crucial steps in ECM mechanisms and offers insights into reactions between organic molecules and silver ions (ESI, Study of device working mechanism, Note S2†). Therefore, to gain insights into the discrepancies in electrical performance exhibited by the two distinct types of molecular memristors rooted in the ECM mechanism, we conducted spectroelectrochemical studies tailored to organic solution systems. The testing setup is shown in Fig. 3b (ESI, Study of device working mechanisms, Notes S4 and S5†).

An electrochemical workstation is utilized to apply bias to the system, while UV-vis is concurrently employed to continuously acquire spectral data. In the inert three-electrode system (ESI, Study of device working mechanisms, Note S6†), it is noteworthy that, upon completion of the scanning process, TZ-1 reverts to its initial value, whereas TZ-2 fails to do so (Fig. S9a, b, and S10†). This evidences that TZ-2 undergoes “electrical aging” under the applied bias voltage. It can be deduced that the TZ-1-based memristor demonstrates a stable HRS (Fig. S11†), whereas the TZ-2-based memristor exhibits fluctuating HRS under the action of voltage bias, which is consistent with the TS behaviors mentioned in Fig. 2e and f.

In addition, in the active three-electrode system (ESI, Study of device working mechanisms, Note S7†) (Fig. S9c and d†), during the voltage bias scanning, the intensity of TZ-1 absorption (Fig. 3c, left) begins to increase at the 1.5 min time point, while the intensity of TZ-2 absorption (Fig. 3c, right) starts to rise at the 2 min time point. This observation is consistent with the low *V*_SET_ property of TZ-1, as demonstrated in Fig. 2e and f. To gain a more precise understanding of the response characteristics of TZ-1 and TZ-2 to an applied voltage, we measured the switching time (HRS transition to LRS duration). By subjecting the same voltage pulse amplitude and width, we found that TZ-1 exhibited a switching time of 60 ns, whereas TZ-2 had a switching time of 110 ns. These findings further substantiate that TZ-1 exhibits a shorter switching time and a low *V*_SET_.

The electron injection energy barriers (difference between Ag work function and LUMO) for TZ-1 and TZ-2 are estimated to be 1.26 eV (Fig. 3e). Despite TZ-2 having the same electron injection energy barrier as TZ-1, it is TZ-1 that first exhibits a change in electrical conductivity. This phenomenon suggests that TZ-1 plays an accelerating and facilitating role in the oxidation of Ag atoms to Ag^+^, thereby causing a faster alteration in electrical conductivity (that is, a low *V*_SET_). Ultimately, this verifies that TZ-1 is important in facilitating the redox process in the ECM mechanism.

We also observed a clear correlation between spectral variation and bias voltage. In the inert electrode system, after completing the voltage scan, the absorption spectrum of TZ-2 did not revert to the initial state, confirming that an irreversible reaction and unstable HRS occurred due to the voltage scan. Conversely, in the active electrode system using an Ag electrode, TZ-1 exhibited a change in absorbance before TZ-2, and the switching time was less than that for TZ-2, definitively indicating that the TZ-1-based memristor has a lower *V*_SET_ compared to the TZ-2-based memristor. This finding is consistent with previously reported TS behavior.

### Verification of the stability of the TZ-1 memristor

**Fig. 4:**
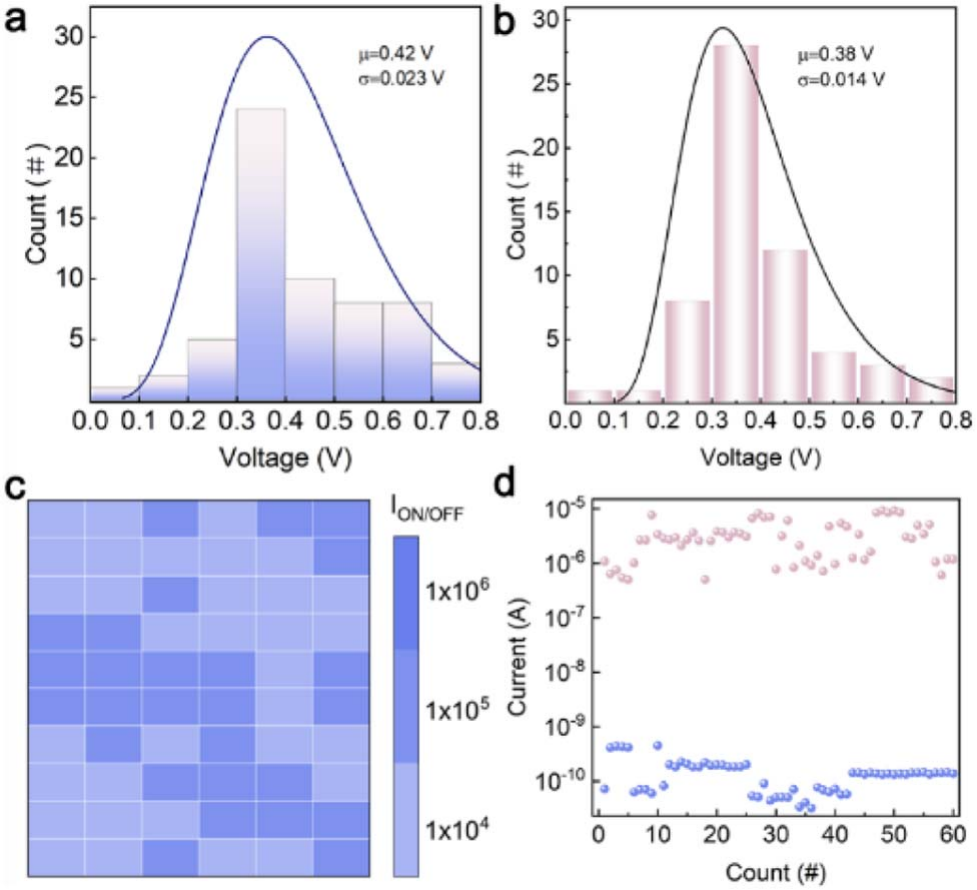
Cross-cycle consistency and long-term durability of memristors. (a) Statistical distribution graph of *V*_SET_ extracted from 60 *I*–*V* curves for a fresh TZ-1-based memristor. (b) Statistical results for *V*_SET_ extracted from 60 *I*–*V* curves for a TZ-1-based memristor stored in an atmospheric environment for 20 weeks. (c) *I*_ON/OFF_ ratio extracted from 60 *I*–*V* curves for a TZ-1-based memristor stored in an atmospheric environment for 20 weeks. (d) Cycling test results for a TZ-1-based memristor stored in an atmospheric environment for 20 weeks.

Before mimicking nociceptor behaviors, it was necessary to verify the RS reliability of TZ-1. Therefore, we conducted a test on the device RS properties, focusing on the parameters of *V*_SET_ and *I*_ON/OFF_. To assess the cycle-to-cycle variation, 60 consecutive *I*–*V* cycles were evaluated to extract the corresponding *V*_SET_ values (Fig. 4a); the average value is 0.42 V. Furthermore, the *I*_ON/OFF_ values were extracted, and the results, as shown in Fig. S12,† also demonstrate the uniformity of the device. To assess device-to-device uniformity, 12 devices were randomly selected for recording *I*–*V* curves (Fig. S13†). Large-scale fabrication and testing of the device were also carried out, as shown in Fig. S14.† To validate the key characteristics of cross-cycle consistency and long-term durability of organic memristors in simulating nociceptor systems, we conducted 60-cycle endurance tests on devices that had been stored in an atmospheric environment for over 20 weeks. Statistical data reveal that *V*_SET_ of the devices exhibit a notably narrow distribution (with a mean value *μ* = 0.35 V) (Fig. 4b). A two-dimensional heatmap constructed based on a 0.05 V read voltage demonstrates a uniform spatial distribution of the *I*_ON/OFF_ ratio within the range of 10^5^ (Fig. 4c). Compared to the fresh device, there is a slight decay in the on–off ratios, but they remain at a relatively high level. The cycling test of the TZ-1 device was carried out and the result was shown in Fig. 4d. The experimental results indicate that the devices can maintain excellent cycling stability and parameter consistency even after long-term storage, providing crucial experimental evidence for the biomimetic simulation of nociceptors in biological neural systems using organic memristors.

### Mimicking nociceptor behaviors

**Fig. 5:**
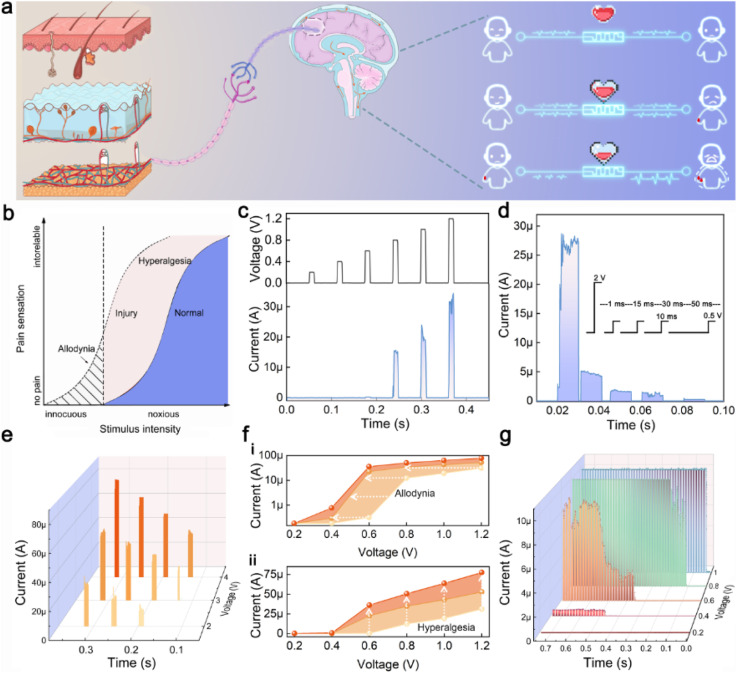
Simulation of four characteristics of nociceptors. (a) Schematic of the anatomical organization of the nociceptor system and the visual representation of pain perception. (b) After injury, pain sensation can be changed, resulting in allodynia or hyperalgesia. (c) The simulation of the “threshold” feature. The response current occurs only when the input voltage exceeds the threshold value. Input voltage, amplitude: 0.2–1.2 V; width: 10 ms, interval: 50 ms. (d) The simulation of the “relaxation” feature. After a normal nociceptor is injured by a strong stimulus, its response gradually relaxes back to the initial state over time as the stimulus subsides. Input voltage, amplitude (strong stimulus): 2 V, amplitude (weak stimulus): 0.5 V; width: 10 ms, interval: 1 ms–15 ms–30 ms–50 ms. (e) The simulation of the “sensitization” feature. Effective pulses, amplitude: 2, 3 and 4 V, 10 ms; pulse trains, amplitude: 0.2–1.2 V; width: 10 ms, interval: 50 ms. (f) The corresponding maximum response currents extracted from (e) on logarithmic scale (i) and linear scale (ii). Input voltage, amplitude: 0.2–1.2 V; width: 10 ms, interval: 50 ms. (g) The simulation of the “no adaptation” feature. Input voltage, amplitude: 0.2–1 V; width: 10 ms, interval: 5 ms, pulse number: 60.

Biological nociceptors are primary sensory neurons that perceive harmful stimuli and transmit pain signals to the brain *via* neurons, eliciting corresponding responses. For example, when the harm exceeds a threshold, a painful expression is exhibited (Fig. 5a). Nociceptors are typically situated at the nerve terminals in biological tissues, and upon stimulation by painful stimuli, they release neurotransmitters, resulting in the depolarization of the postsynaptic neuron's membrane potential.^3^ When the depolarization potential exceeds the threshold value (*V*_th_), pain signals are released. After releasing a pain signal, a biological nociceptor undergoes a hyperpolarization process, which is considered a “relaxation” process. During the recovery period, to protect the organism from further damage at the site of injury, the “sensitization” feature is activated (Fig. 5b).

In biological nociceptors, the sensitization phenomenon usually manifests as “allodynia” and “hyperalgesia”. Theoretically, allodynia can be defined as a painful response to a no-injury stimulus, which does not exceed the threshold of the nociceptors. Hyperalgesia can be defined as an irregular painful response (*i.e.*, exceeding the normal pain response) to an injury-causing stimulus. Finally, unlike other receptors, which tend to gradually adapt to ongoing signal stimuli, nociceptors do not exhibit such adaptation, which is the “no adaptation” property. The aforementioned characteristics constitute the essential attributes of biological nociceptors. Memristors that operate based on the ECM mechanism can exhibit analogous behaviors to those of nociceptors. Leveraging their excellent consistency and uniformity, TZ-1 devices can be applied to effectively emulate nociceptors for realizing these four functions.

To simulate the “threshold” feature, a series of voltage pulses (amplitude: 0.2–1.2 V) with a step of 0.2 V were applied to the TZ-1 TSM (Fig. 5c). The memristor responds only at a pulse value exceeding 0.8 V, demonstrating the “threshold” feature, which depends on the stimulus intensity. Additionally, the pain (or discomfort) response can also be triggered by increasing the pulse width at a relatively low stimulus intensity (0.6 V) (Fig. S15†). Fig. S16† illustrates the threshold behavior of TZ-2 in simulating a nociceptor. The elevated HRS results in the generation of electrical signals even at low voltages, which may compromise the accuracy of pain signal detection. This further confirms that TZ-2 is not suitable for the simulation of nociceptors.

For the simulation of the “relaxation” characteristic, after an initial strong stimulus (2 V, 10 ms) induced a pain signal, subsequent sub-threshold weaker stimuli (0.5 V, 10 ms) were applied. The results in Fig. 5d indicate that the pain signal response can fully return to its initial state, demonstrating the “relaxation” characteristic.

During the “relaxation” process, nociceptors lower their threshold to shield the injured tissue from further damage, a phenomenon regarded as the “sensitization” characteristic. To study the “sensitization” characteristics, different effective pulses were applied to the TZ-1-based artificial nociceptor to induce varying degrees of tissue damage and pain responses. Subsequently, pulse trains (0.2 to 1.2 V, step size: 0.2 V, pulse width: 10 ms) were used to record the current responses. The results in Fig. 5e show that the stimuli with different intensities produce distinct responses. Specifically, Fig. 5f exhibits the averaged response currents extracted from Fig. 5e on both logarithmic (Fig. 5f(i)) and linear (Fig. 5f(ii)) scales. With the increasing severity of the injury, the threshold drops, and the current response intensifies, suggesting that nociceptors with greater damage become more sensitive to external stimuli, that is, “allodynia” and “hyperalgesia” behaviors.

Fig. 5g illustrates the response currents to pulse trains with varying amplitudes. It is observed that as the pulse value increases, the nociceptor reaches its threshold more quickly, and once this threshold is exceeded, it continues to generate outputs. This behavior demonstrates the “no adaptation” nature and threshold property of the artificial nociceptor, which differs from other types of receptors.

In this section, we harnessed the designability of small organic molecules to manipulate and enhance device performance, achieving successful application in the neuromorphic domain.

## Conclusions

This study investigated the working mechanisms of memristors by employing spectroelectrochemical techniques. Through meticulous molecular design, TZ-1 and TZ-2 with equivalent electron transport barriers were synthesized and utilized as the active layer in memristors. The experimental data reveals a high degree of alignment between the electrical characteristics and the outcomes derived from spectroelectrochemical analysis. Repeated validation between electrochemical and spectroscopical results affirmed the applicability of spectroelectrochemical techniques in probing memristor mechanisms. Additionally, our investigation shows that the TZ-1-based TSM demonstrates remarkable performance, including low device variability (*V*_SET_ and *I*_ON/OFF_ ratio) and pioneering application as nociceptors. This achievement not only diversifies the application landscape of organic small-molecule memristors but also verifies the application prospects of molecular design in the semiconductor field. Customizing the active layer can significantly reduce power consumption and optimize device performance.

## Author contributions

JiYu Zhao: conceptualization, investigation, writing – original draft, methodology; Kun Liu: data curation, software, resources; Wei Zeng: data curation, formal analysis; Zherui Zhao: visualization; Zhuo Chen: validation; Yifan Zheng: validation; Wen-Min Zhong: software; Su-Ting Han: investigation; Guanglong Ding: writing – review & editing; Ye Zhou: writing – review & editing, funding acquisition; Xiaojun Peng: supervision, funding acquisition.

## Conflicts of interest

The authors declare no conflicts of interest.

## Supplementary Material

SC-OLF-D5SC02710A-s001

## Data Availability

The data supporting the findings of this study are available from the authors upon reasonable request.
