# Correction: Unveiling the switching mechanism of robust tetrazine-based memristive nociceptors *via* a spectroelectrochemical approach

**DOI:** 10.1039/d5sc90148k

**Published:** 2025-07-04

**Authors:** JiYu Zhao, Kun Liu, Wei Zeng, Zhuo Chen, Yifan Zheng, Zherui Zhao, Wen-Min Zhong, Su-Ting Han, Guanglong Ding, Ye Zhou, Xiaojun Peng

**Affiliations:** a College of Materials Science and Engineering, Shenzhen University Shenzhen 518060 P. R. China; b Institute for Advanced Study, Shenzhen University Shenzhen 518060 P. R. China yezhou@szu.edu.cn; c State Key Laboratory of Fine Chemicals, Frontiers Science Center for Smart Materials, Dalian University of Technology Dalian 116024 P. R. China pengxj@dlut.edu.cn; d Department of Applied Biology and Chemical Technology, Research Institute for Smart Energy, The Hong Kong Polytechnic University Hung Hom Hong Kong SAR 999077 P. R. China; e State Key Laboratory of Radio Frequency Heterogeneous Integration, Shenzhen University Shenzhen 518060 P. R. China dinggl@szu.edu.cn

## Abstract

Correction for ‘Unveiling the switching mechanism of robust tetrazine-based memristive nociceptors *via* a spectroelectrochemical approach’ by JiYu Zhao *et al.*, *Chem. Sci.*, 2025, https://doi.org/10.1039/d5sc02710a.

The authors regret that there is a typo in the current ON/OFF ratio given in the Abstract. The high current ON/OFF ratio is ∼10^6^.

The Royal Society of Chemistry apologises for these errors and any consequent inconvenience to authors and readers.

