# Supplementary material for: Unveiling the switching mechanism of robust tetrazine-based memristive nociceptors via a spectroelectrochemical approach
Source: Chem Sci. 2025 Jun 3;16(27):12362–71. doi: 10.1039/d5sc02710a (PMC12150463; doi:10.1039/d5sc02710a)
Supplement: SC-016-D5SC02710A-s001 [file SC-016-D5SC02710A-s001.pdf]

Supplementary Information (SI) for Chemical Science.
This journal is © The Royal Society of Chemistry 2025

Supporting Information

Unveiling the switching mechanism of robust tetrazine-based memristive nociceptors via a spectroelectrochemical approach

JiYu Zhao,[a,b,c] Kun Liu,[c] Wei Zeng,[b] Zhuo Chen,[b] Yifan Zheng,[b] Zherui Zhao,[b] Wen-Min Zhong,[b] Su-Ting Han,[d] Guanglong Ding,*[e] Ye Zhou*[b] and Xiaojun Peng*[a,c]

[a] College of Materials Science and Engineering, Shenzhen University, Shenzhen 518060, P. R. China. E-mail: pengxj@dlut.edu.cn

[b] Institute for Advanced Study, Shenzhen University, Shenzhen 518060, P. R. China. E-mail: yezhou@szu.edu.cn

[c] State Key Laboratory of Fine Chemicals, Frontiers Science Center for Smart Materials, Dalian University of Technology, Dalian 116024, P. R. China.

[d] Department of Applied Biology and Chemical Technology and Research Institute for Smart Energy, The Hong Kong Polytechnic University, Hung Hom, Hong Kong SAR 999077, P. R. China.

[e] State Key Laboratory of Radio Frequency Heterogeneous Integration, Shenzhen University, Shenzhen 518060, P. R. China. E-mail: dinggl@szu.edu.cn

1. Materials and Synthesis

1.1. Materials

5-Bromothiophene-2-carbonitrile (>99.8%), 2-Cyanothiophene (>99.8%), Sulfur (>99.8%), Sodium Nitrite (>99.8%), Potassium Carbonate (>99.8%), and Sodium Sulfite (>99.8%) purchased from Energy Chemical. 4-Triphenylborane-boronic acid (>99.8%), Tetrakis (triphenylphosphine) palladium (>99.8%), 4,4'-Dimethoxy-4''-triphenylborane-boronic acid (>99.8%), and (4-(Di-p-tolylamino)phenyl)boronic acid (>99.8%) were purchased from Bide Pharmatech Ltd. Hydrazine hydrate (>60%), Tetrahydrofuran (THF) (>99.8%), Dichloromethane (DCM) (>99.8%).Acetone (purity >99.8%) and anhydrous ethanol were sourced from Dalian Bonuo Biochemical Reagent Factory. All of the aforementioned chemical raw materials and reagents were utilized directly without any prior purification upon purchase.

1.2. The synthesis and characterization of TZ molecules

3, 6-bis (5-bromothiophen-2-yl) -1,2,4, 5-tetrazine (TZ): The mixture of anhydrous ethanol solution (5 ml), including the hydrazine monohydrate (2.50 mL, 50 mmol, 1 eq), 5-bromothiophene-2-methyl nitrile (2.35 g, 12.5 mmol, 2 eq) and sulfur (0.24 g, 7.5 mmol, 0.5 eq). Then the mixture refluxed at 80 ℃. The reaction process monitored by TLC. After the reaction, the ice bath for 1h obtained the precipitation that rapid filtration and ethanol was washed obtain the crude intermediate product (2.10 g, 5.17 mmol, 1 eq). Mixture the intermediate product with sodium nitrite (1.07 g, 15.51 mmol, 3 eq) aqueous solution (100 mL) in dichloromethane (10 mL). At the 0 ℃ slow drop the acetic acid to the mixture,while stirred at room temperature to reaction and by TCL monitored the reaction process. When the after the reaction, the just removed dichloromethane solvent through rotary evaporation. An ice bath cools through the vacuum filtration obtained an orange-yellow solid wash by cold

water and dry under vacuum. The crude product (dichloromethane: petroleum ether 1:1) was purified by silica gel by column chromatography, and Tz (1.87 g, yield 37%) was obtained.

Nuclear magnetic resonance (NMR) of TZ: 1H NMR (500 MHz, Chloroform-d) δ8.02(d, J=4.0 Hz, 2H), 7.24(d, J=4.1 Hz, 2H).

MS(APCI) of TZ: $C_{10}H_4Br_2N_4S_2([M+H]^-)$, Exact Mass: 403.8224, Test Value: 403.8

4,4'- ((1,2,4, 5-tetrazine-3, 6-diyl) bis (Thiophen-5, 2-diyl)) bis (N, n-bis (4-methoxyphenyl) aniline) (TZ-1):

TZ (0.3 g, 0.74 mmol), Triphenylamine 4, 4-dimethoxy-4-borate (0.52 g, 1.49 mmol) ((4-(di-p-tolueno-amino) phenyl) boric acid, 0.48 g, 1.49 mmol), tetrtriphenylphosphine palladium (0.07 g, 0.06 mmol), and Potassium carbonate (0.3 g, 2.22 mmol) was added to a mixture of tetrahydrofuran (20 mL) and deionized water (10 mL), reaction at nitrogen protected and heated to 95 ℃ reflux. The reaction process was monitored by TLC and the reaction temperature was cooled to room temperature after the reaction. Through the vacuum filtration obtained the red solids, which washed by cold water and a small amount of toluene, under vacuum to dry. The crude product (dichloromethane: petroleum ether 1:1) was purified by silica gel by column chromatography to obtain TZ-1(0.49 g, 91% yield) (TZ-2, 0.53 g, 91% yield).

NMR of TZ-1: 1H NMR (500 MHz, Chloroform-d) δ8.19(d, J=3.9 Hz, 2H), 7.53(d, J=8.6 Hz, 4H), 7.34(d, J=4.0 Hz, 2H), 7.15–7.10(m, 8H), 6.95(d, J=8.7 Hz, 4H),6.92–6.86(m, 8H),3.84(s, 12H).

MS(APCI) of TZ-1: $C_{50}H_{40}N_6O_2S_2$ ($[M+H]^+$), Exact Mass: 852.2552, Test Value: 852.2.

4,4'- ((1,2,4, 5-tetrazine-3, 6-diyl) bis (Thiophen-5, 2-diyl)) bis (N, n-di-p-tolylaniline) (TZ-2):

TZ (0.3 g, 0.74 mmol), 4-(di-p-tolueno-amino) phenyl) boric acid, 0.48 g, 1.49 mmol, tetrtriphenylphosphine palladium (0.07 g, 0.06 mmol), and Potassium carbonate (0.3 g, 2.22 mmol) was added to a mixture of tetrahydrofuran (20 mL) and deionized water (10 mL),

reaction at nitrogen protected and heated to 95 ℃ reflux. The reaction process was monitored by TLC and the reaction temperature was cooled to room temperature after the reaction. Through the vacuum filtration obtained the red solids, which washed by icy water and a small amount of toluene, under vacuum to dry. The crude product (dichloromethane: petroleum ether 1:1) was purified by silica gel by column chromatography to obtain TZ-2, 0.53 g, 91% yield.

NMR of TZ-2: 1H NMR (500 MHz, Chloroform-d) δ8.16(d, J=4.0 Hz,2H), 7.53–7.48(m, 4H), 7.32(d, J=4.0 Hz, 2H), 7.25(s, 0H), 7.10(d, J=8.3 Hz, 8H), 7.07–6.99(m, 8H),2.33(s, 12H).

MS(APCI) of TZ-2: $C_{50}H_{40}N_6O_2S_2$ ($[M+H]^+$), Exact Mass: 788.2756, Test Value: 789.3.

1.3. Energy band gaps of TZ molecules:

The band gaps were obtained based on the UV−Vis results of TZ molecules (Figure S2). The onset of the absorption peaks of the TZ-1 and TZ-2 films are determined to be 574 and 560 nm (λ_{edge}), from which the optical bandgap (E_{eg}) was calculated to be approximately 2.16 and 2.21 eV according to the Equation S1, respectively.

$$E_{eg} = h_c/\lambda_{edge} = 1240/\lambda_{edge} \quad \text{(S1)}$$

$$E_{HOMO} = -\left[E_{OX(onset) vs\ Ag/AgNO3} + 4.8 - E_{Ferrocene}\right] \quad \text{(S2)}$$

$$E_{LUMO} = E_{HOMO} - E_{eg} \quad \text{(S3)}$$

The HOMO energy levels of the TZ-1 and TZ-2, calculate by Equation S2, the energy levels of the highest occupied molecular orbital (HOMO) are -5.16 eV and -5.21 eV, respectively. Utilizing Equation S3, the lowest unoccupied molecular orbital (LUMO) energy levels of TZ-1 and TZ-2 have been determined to be -3 eV and -3 eV, respectively.

The band gaps of TZ-1 and TZ-2 obtained from UV−Vis are 2.16 and 2.21 eV (Table 1), respectively, which are consistent with the values obtained from density functional theory (DFT) calculation (Figure S3). A narrower band gap facilitates more efficient intramolecular

charge transfer, thereby enhancing the charge transfer properties of the film. Therefore, theoretically, TZ-1 may exhibit superior charge transfer properties compared to TZ-2, and result in better device electrical behaviors.

2. Device Fabrication and Characterization

Pre-patterned ITO glasses were ultrasonically cleaned sequentially in acetone, ethanol, isopropanol, and deionized water at 50% power for 15 minutes each. After drying under a N_2 flow, the small-molecule active layer was deposited via spin-coating from a 5 mg/mL acetonitrile solution at the speed of 1500 rpm (45 s), followed by annealing at 100 °C (10 min). A 50 nm thick Ag TE was then thermally evaporated through a shadow mask, after which a 15 nm Au capping layer was added to prevent oxidation. The TE dimensions were 210 μm × 50 μm. Atomic force micrographs (AFM) were examined using a BRUKER L01F4C8. The scanning electron microscopy (SEM) images were obtained using a field emission scanning electron microscope (FE-SEM, Zeiss Gemini560). Electrical measurements were conducted using a KEYSIGHT 2902B Semiconductor Device Analyzer in ambient conditions, with the electrical bias applied to the TE while the ITO BE was grounded.

3. Study of Device Working Mechanisms

3.1. Note S1

Gaussian 09W software performed pertinent theoretical calculations on molecular systems employing the b3lyp/6-31g(d) methodology within the framework of density functional theory (DFT). Optimize the molecular structure and simulate the calculation of orbital energies and electrostatic potentials for HOMO-1, HOMO, LUMO, and LUMO+1 of molecules TZ-1 and TZ-2. The positive ESP (green region) is mainly obtained from the triphenylamine electron-

donating group, whereas the negative ESP (red region) is concentrated around the tetrazine electron-attractive group. A significant ESP difference between the intramolecular acceptor and donor facilitates the enhancement of current flow. Additionally, the proximity of electron-rich and electron-deficient regions among molecules promotes efficient electron conduction. Consequently, both types of molecules are ideally suited for applications within the realm of electronic devices.

3.1. Note S2

The RS process of ECM based memristor with Ag anode can be mainly divided into three steps: 1, oxidation of Ag atoms into Ag^+ ions at the interface of anode under the action of positive bias; migration of Ag^+ ions to the cathode within the solid electrolyte under the driven of applied positive electric field; and the reduction of Ag^+ ions into Ag atoms to form the metallic CFs. These processes alter the device resistance and collectively determine its RS behavior and stability.

Specifically, Ag atom oxidation primarily takes place at the anode, influenced by the energy barrier, applied potential and Ag atom oxidation rate difference. The migration of Ag ions, influenced by factors such as the microstructure and defects of the solid electrolyte, is directed towards the cathode under the action electric field. At the cathode, Ag ions accept electrons and are reduced to Ag atoms, which accumulate to form metallic CFs. Given the distinct CV results between TZ-1 and TZ-2, as well as their similar molecular structures, it is plausible that the influence of TZ molecules on oxidation and reduction processes of Ag primarily accounts for the differing electrical behaviors observed in TZ-1 and TZ-2 based memristors.

Spectroelectrochemistry is a field that focuses on studying the electrode/electrolyte interface using spectroscopic techniques. These techniques provide a powerful means to investigate

the chemical and physical processes occurring at the interface between an electrode and its surrounding electrolyte solution. Furthermore, spectroelectrochemical techniques enable the investigation of complex electrochemical processes, such as electron transfer reactions, ion solvation, and the formation of intermediates and products during electrode reactions. This information is crucial for understanding and optimizing the performance of electron devices.

Therefore, to examine the effects of two organic small molecule systems on the RS mechanisms of ECM, especially the oxidation and reduction processes, an extensive analysis was undertaken by employing spectroelectrochemical techniques.

3.3. Note S3

We investigated the device RS mechanism by using spectroelectrochemical techniques. Beforehand, both films and solutions of TZ molecules are characterized through Raman (Figure S4) and XRD (Figure S5). Both Raman and XRD results between TZ-1 and TZ-2 exhibit no discernible differences, indicating the similar existence forms of TZ molecules in both solution and films, that is, disordered state. Therefore, to capture the influence of small molecules more accurately on the RS process, the solution system was chosen.

3.4. Note S4

Conduct research on the mechanism of the TZ based memristors device through spectroelectrochemistry methods.

Spectroelectrochemistry equipment: the UV–Vis absorption spectra of TZ molecule in DCM solutions were measured under the external voltage sweeping. The schematic diagram of test equipment is shown in Figure 3c. It's worth noting that the glass sample pool was designed and customized for facilitating the test.

Inert three-electrode system: of working electrode (Pt), reference electrode (Ag/$AgNO_3$) and counter electrode (Pt).

Active three-electrode system: of working electrode (Ag), reference electrode (Ag/$AgNO_3$) and counter electrode (Pt).

Testing methods: An electrochemical workstation is utilized to apply a specific sweeping voltage to the electrochemical system (from 0 to 1.2 V and then back to 0 V). During this process, the UV–Vis collects spectra to monitor changes in substances during the reaction. This synchronous approach allows for real-time observation of the impact of electrochemical reactions on spectral characteristics.

In the inert electrode system: the electrochemical workstation from 0 to 1.2 V and then back to 0V (scan rates 4.5 mV s^{-1}). Speed of spectral acquisition of 1100 nm min^{-1}.

In the active electrode system: the voltage scan rate is increased to 7 mV s^{-1} and spectral acquisition is risen up to 2200 nm min^{-1} because of the accelerated reaction rate caused by the migration of electrode ions (Ag^{+}s) into the liquid reaction medium.

3.5. Note S5

Under the assistance of customized sample pool, the UV−Vis Infrared (IR) absorption spectrum of inert three-electrode system was collected without the voltage bias as the blank reference (Figure S6). Compared to that in Figure 2c and 2b (800 nm-200 nm), the absorption peaks of the molecules in UV–Vis region with electrodes become are getting broader (Figure S6a), this is caused by the customized sample pool. However, these prominent and broad absorption peaks may obscure the variations in peak values during the RS process, which could complicate the analysis of the RS mechanism. Upon extending the absorption wavelength into the IR region, a series of relatively weak and discrete absorption peaks between 800 and 1500 nm emerge (Figure S6b). These peaks can be attributed to the interface interactions between the metal electrode and the solution. We collected dynamic UV spectra (ranging from 1500 to 400

nm, covering the molecular absorption peaks: at 498 and 487 nm) for both inert and active electrode systems, and the results are shown in Figure S7.

Considering the interfacial oxidation and reduction processes in practical ECM memristors, we propose that analysing the variation of these IR absorption peaks under external voltage sweeps is crucial for understanding the role of TZ molecules in the RS process.

3.6. Note S6

In inert three-electrode system, the applied voltage bias undergoes a gradual increase from 0 to 5 min (0-1.2 V), followed by a gradual decrease from 5 to 9 min (1.2-0 V). The spectroelectrochemistry results in Figures S7(a and b) and S8 indicates that the absorbance of TZ-1 increases at the 4 min time point, followed by a decrease at the 5 min time point. Subsequently, the absorbance of TZ-1 returns to its initial value (corresponding to the absorbance recorded at the 1-min time point) by the 8-min time point, indicating that TZ-1 does not exhibit “electrical aging” under the applied bias. In contrast, TZ-2 shows a decrease in absorbance at the 6-min mark and does not recover to its initial state by the end of the test (at the 9 min mark). This suggests that TZ-2 experiences “electrical aging” when subjected to the same applied bias voltage, potentially leading to a variation of resistance state. Consequently, based on the spectroelectrochemistry results obtained from an inert three-electrode system, it can be deduced that the TZ-1 based memristor demonstrates a stable HRS, whereas the TZ-2 based memristor exhibits fluctuating HRS under the action of voltage bias. These analyses and inferences align with the previously discussed TS behaviour (Figure 2e and 2f, and Figure S9).

3.7. Note S7

In the active electrode system, the applied voltage bias undergoes a gradual increase from 0 to 3 min (0-1.2 V), followed by a gradual decrease from 3 to 6 min (1.2-0 V) (Figure 3C, Figure

S7c and S7d). The spectroelectrochemistry results in Figures 3c and Figure S7c indicate that the absorbance of TZ-1 begins to increase at the 1.5-min time point, reaches its maximum absorption at 3 min, and gradually decreases from 3 to 6 min. For TZ-2, the absorbance starts to rise at the 2 min time point, reaching its maximum absorption at 3 min with the appearance of a new broad peak at 965 nm, which persists until the end of the experiment. This suggests the formation of new substances from TZ-2 under applied voltage, likely due to the complexation of electrically aged TZ-2 with Ag^+, consistent with observations from the inert electrode system. Neither solution system's absorbance returns to its initial state by the end of the test, which can be attributed to the migration and random dispersion of Ag^+s within the solution.

Supporting Figures and Tables

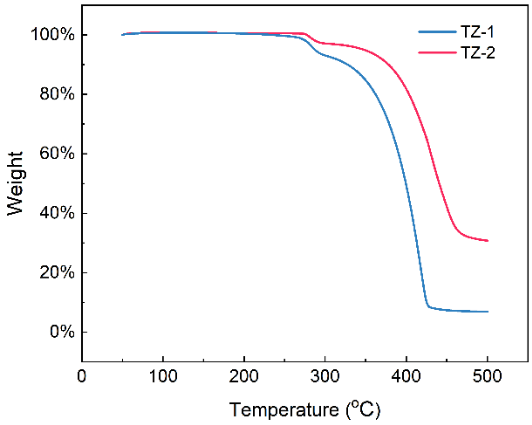

Figure S1. TGA curves for TZ molecules.

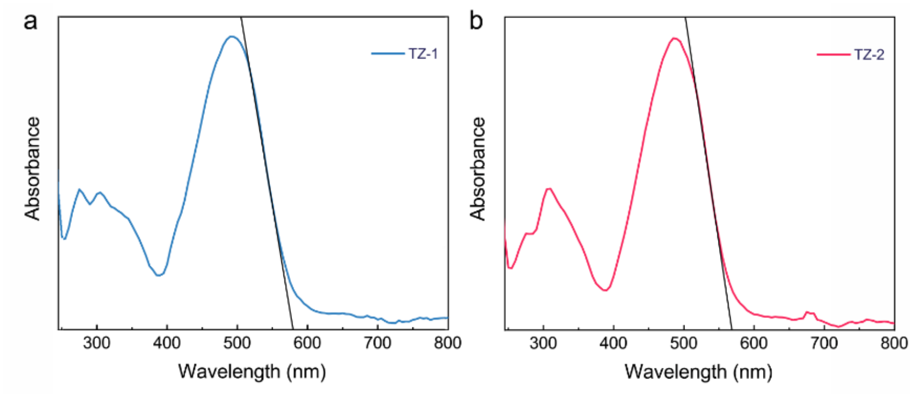

Figure S2. UV−Vis absorption spectra of TZ-1 (a) and TZ-2 (b) films.

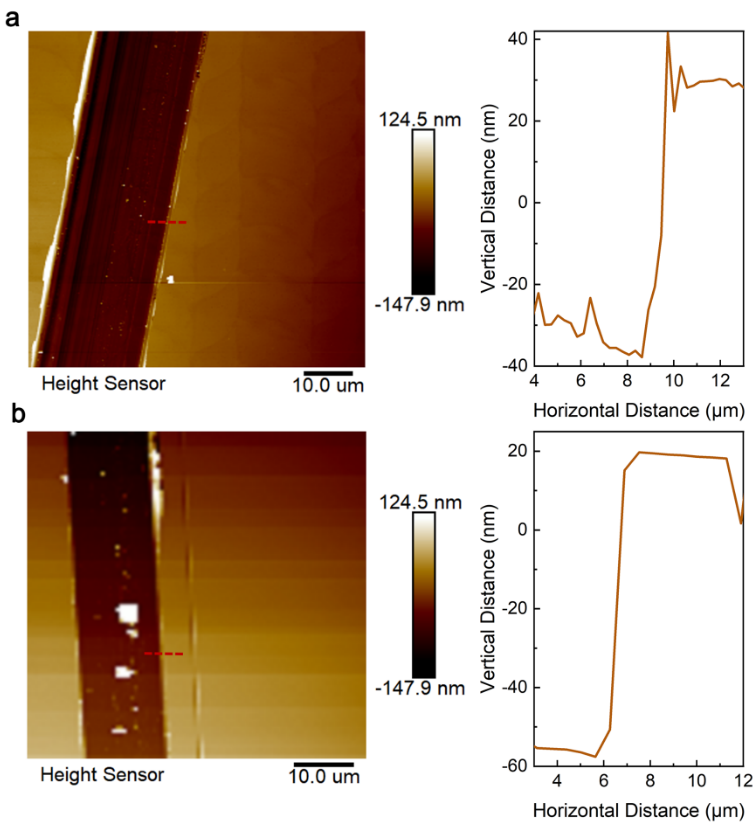

Figure S3. The AFM images and corresponding thickness curves of (a) TZ-1 and (b) TZ-2 films.

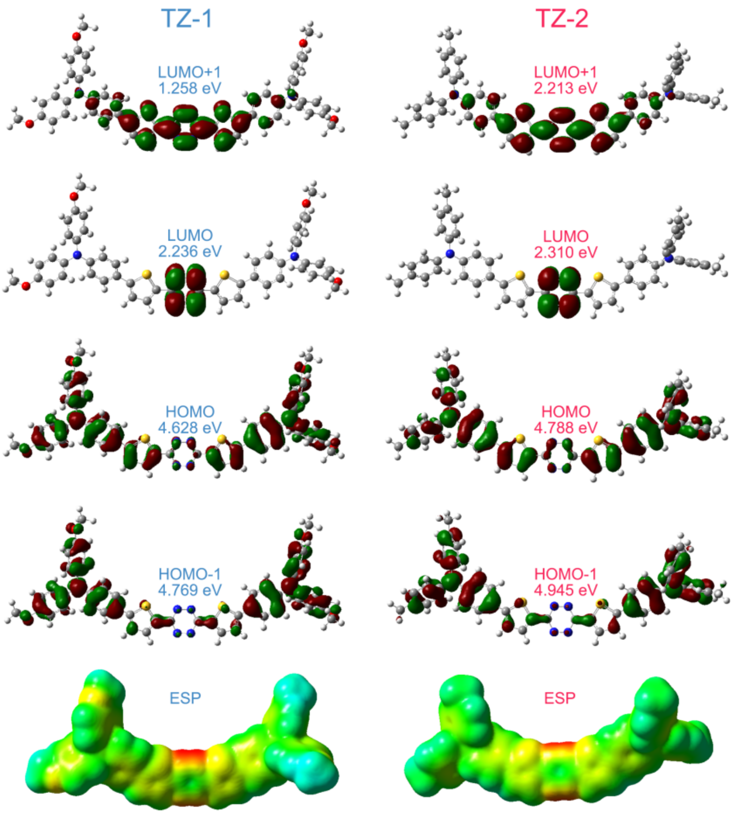

Figure S4. Molecular orbital energy levels and ESP map(bottom) of TZ-1/2 by DFT.

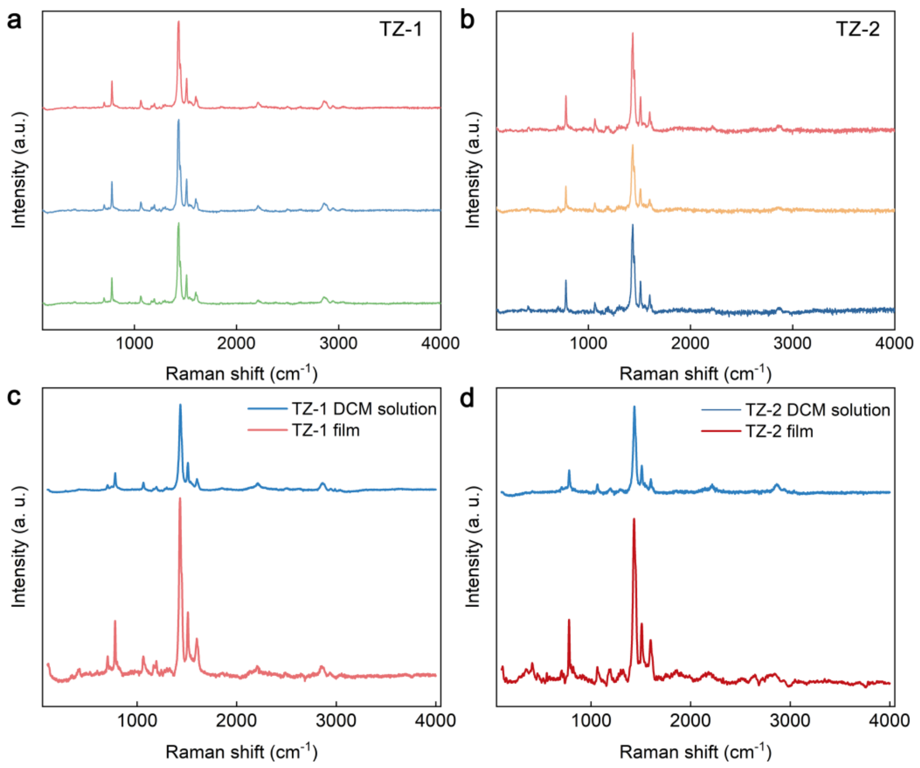

Figure S5. (a-b) Randomly select three positions on TZ-1 and TZ-2 to conduct Raman characterization, thereby proving the uniformity of their thin films. (c-d) The Raman spectra of TZ-1 (c) and TZ-2 (d) in DCM solutions and films.

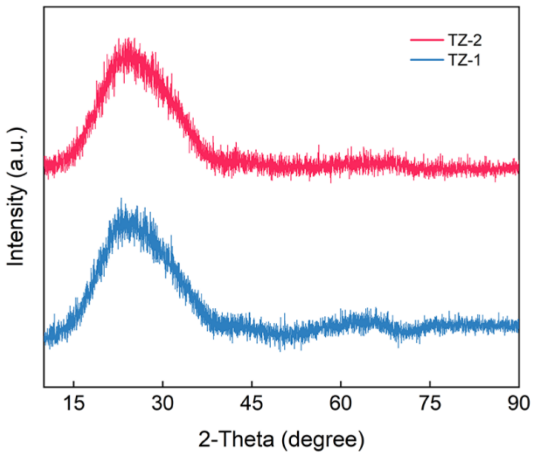

Figure S6. XRD patterns of TZ-1 and TZ-2.

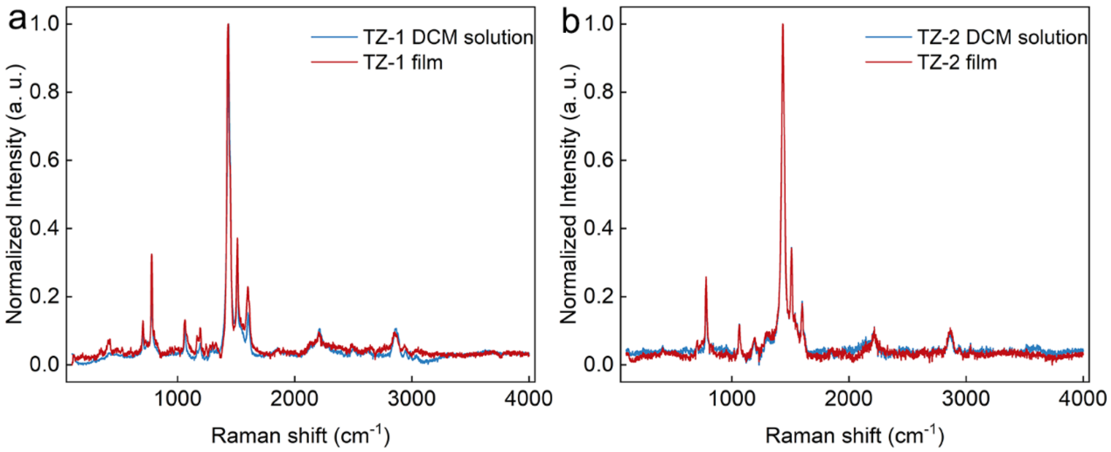

Figure S7. Raman spectra variation of solution and film in all characteristic peak at full spectrum range. (a) TZ-1 variation was 4.5%; (b) TZ-2 variation was 3%.

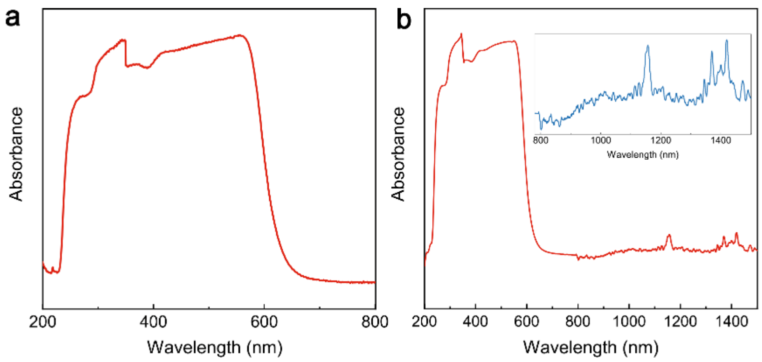

Figure S8. The UV−Vis (a, 800-200 nm) and UV−Vis−IR (b, 1500-200 nm) absorption spectra of DCM solution obtained in the inert three-electrode system under the assistance of specially designed cuvette.

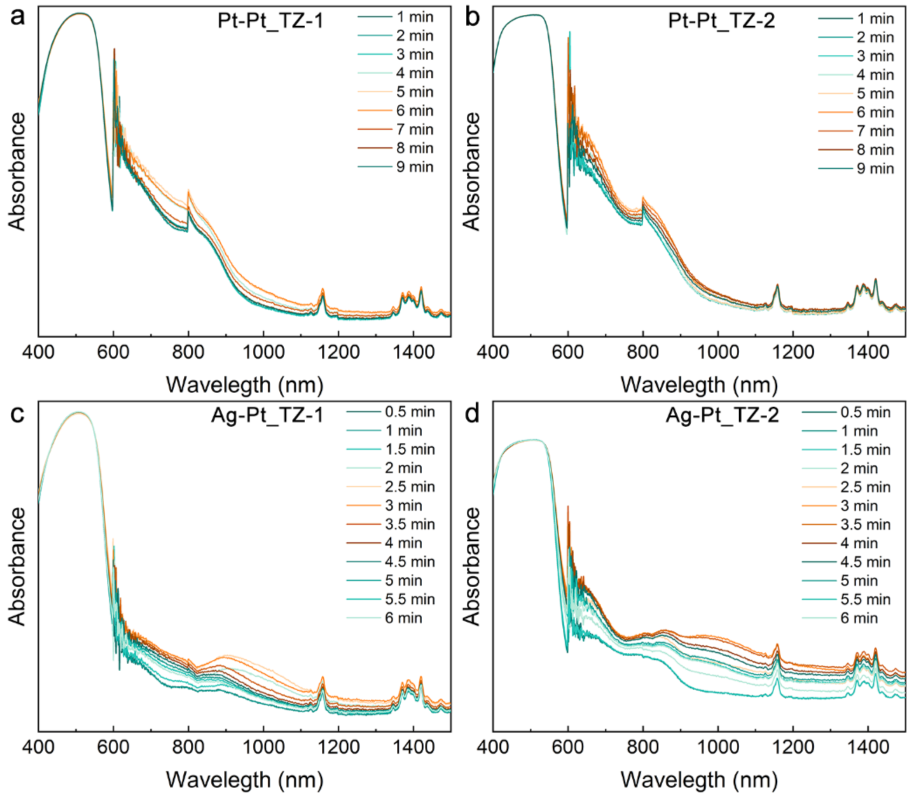

Figure S9. The two-dimensional spectroelectrochemical diagrams of TZ-1 and TZ-2 in inert and active electrode systems, with a test range of 1500-400 nm, encompassing the absorption peaks of compounds TZ-1 and TZ-2 themselves at 498 and 487 nm, respectively.

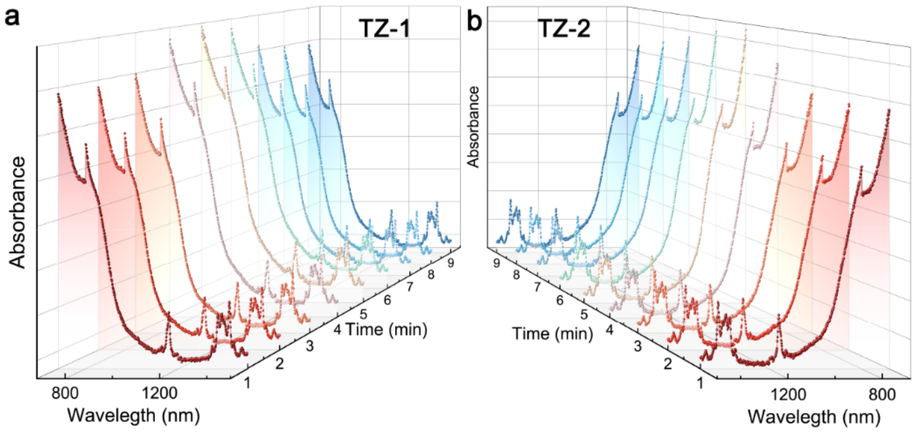

Figure S10. The spectroelectrochemical diagrams of TZ-1 (a) and TZ-2 (b) in inert electrode systems are presented, only the wavelength range of 1500-700 nm is analyzed in the main text.

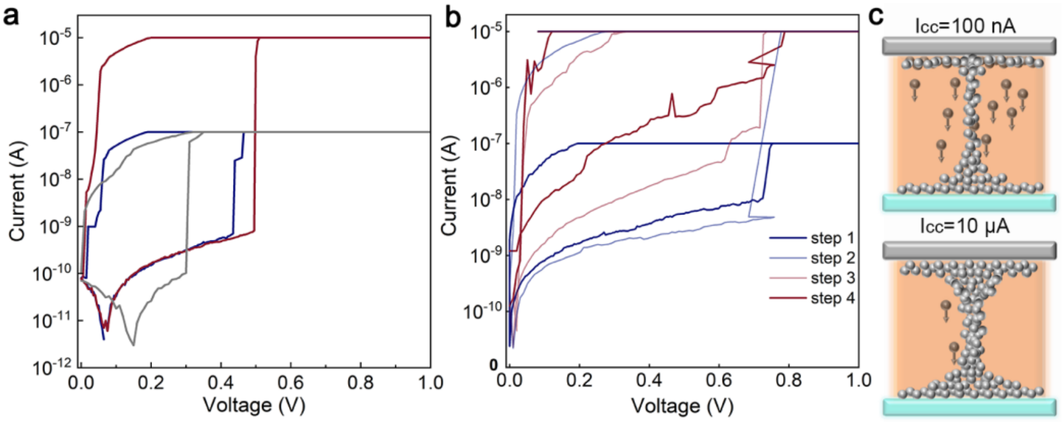

Figure S11. The I-V curves of TZ-1 (a) and TZ-2 (b) at I_{cc}=100 nA and 10 μA, respectively; I_{cc} at Step 1 (Initial State) is 100 nA; I_{cc} at Step 2 is 10 μA; I_{cc} at Step 3 is 10 μA; as depicted in the figure, after I_{cc} is repeatedly scanned several times from 10 μA (Step 2) to reach Step 4, the I-V curves differ by more than one order of magnitude, confirming that TZ-2 undergoes a transformation during multiple cycles, while TZ-1 exhibits stable properties. (c) The illustration of the filament.

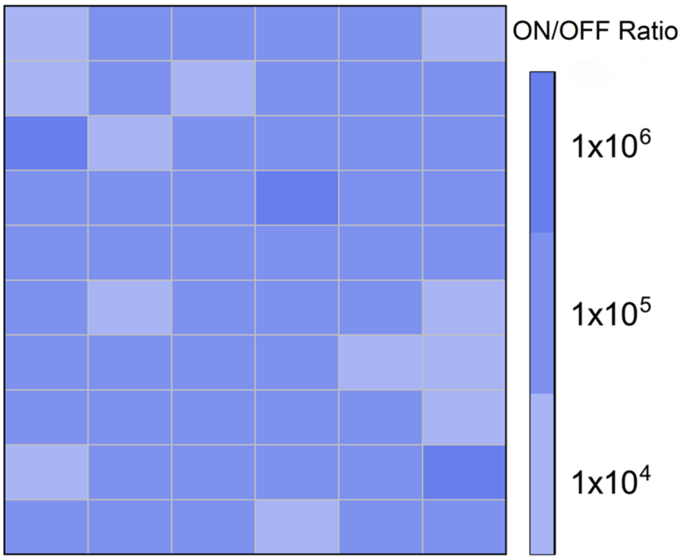

Figure S12. The statistical distribution graph of $I_{ON}/_{OFF}$ ratio extracted from 60 I-V curves of fresh TZ-1 memristor.

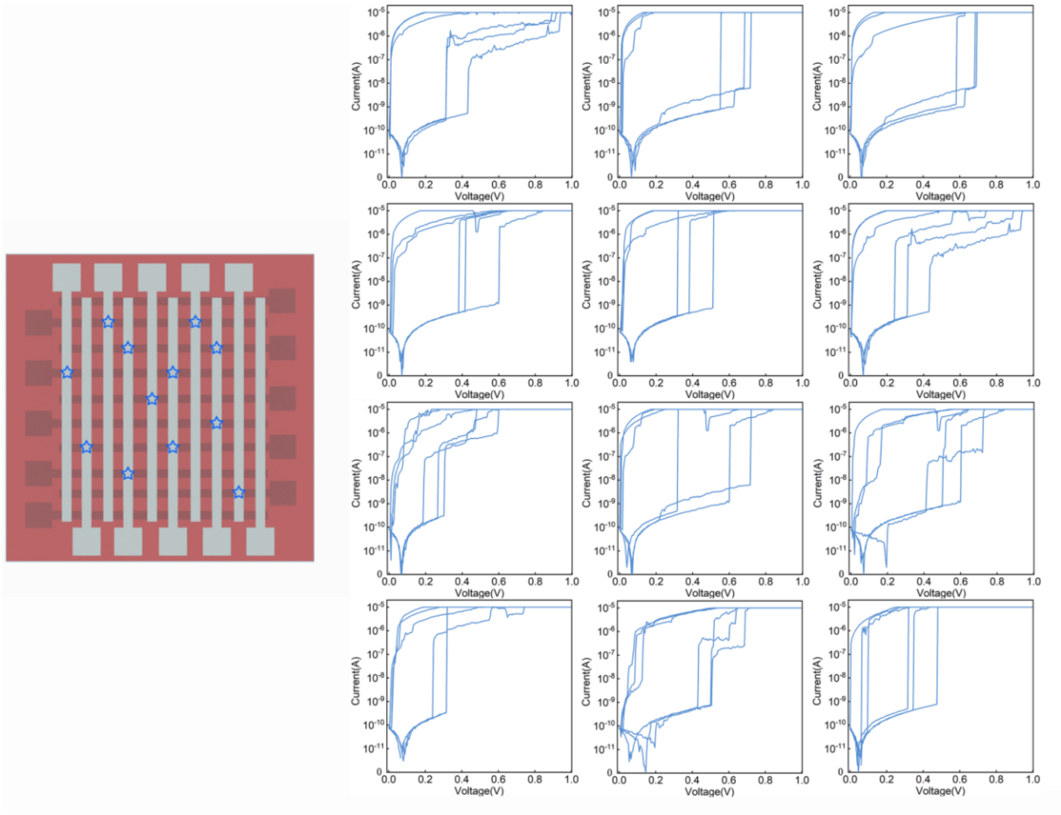

Figure S13. The I-V curves of the random TZ-1-based memristor.

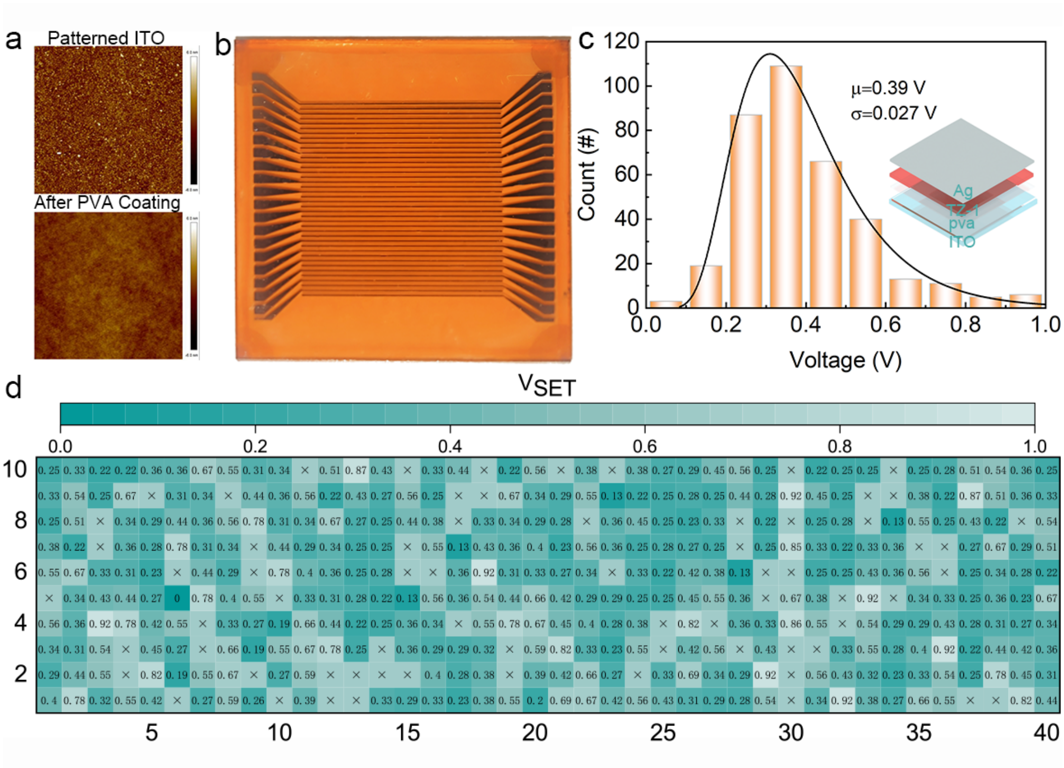

Figure S14. (a) AFM surface topography analysis: (Top) Patterned ITO electrode with a surface roughness (RMS) of 1.7 nm; (Bottom) Surface morphology significantly flattened after spin-coating a PVA film, with the roughness reduced to 0.67 nm. Scale bar: 3 μm. (b) Optical image of the 10 × 40 array. (c) Histogram of the statistical distribution of V_{SET} for 359 memory devices. (d) Distribution of V_{SET}.

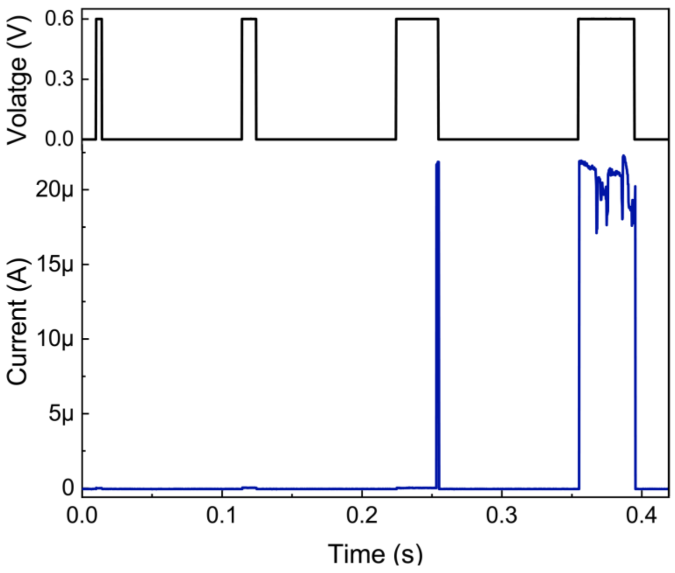

Figure S15. The threshold property of TZ-1-based TSM under fixed pulse amplitude (0.6 V) and different pulse width (2 ms, 5 ms, 10 ms, 20 ms).

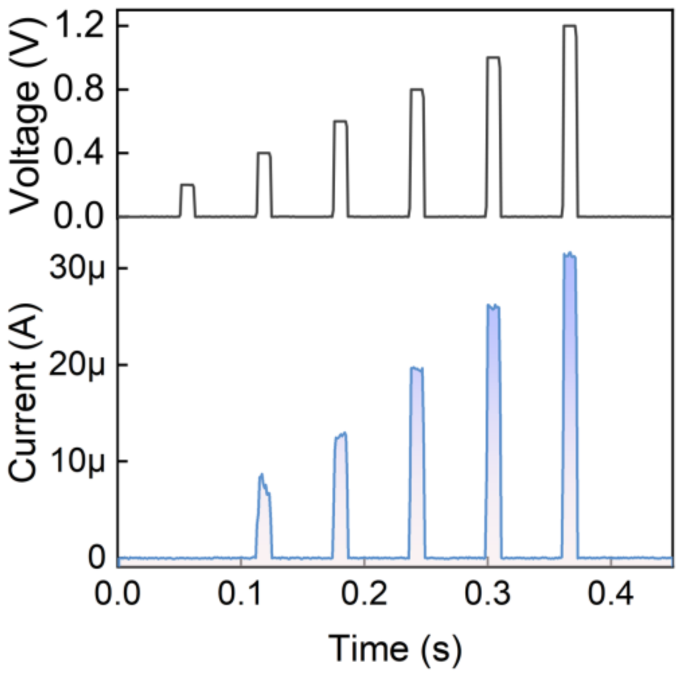

Figure S16. Response currents of artificial nociceptors “thershold” characteristic based on TZ-2 memristors at different pulse amplitudes (0.2−1.2 V) (pulse width, 1 ms; interval, 10 ms).

Table 1. The HOMO, LUMO and energy band gap (Eg) of TZ-1 and TZ-2 obtained from UV−Vis and CV results.

Molecular	HOMO (eV)	LUMO (eV)	Eg (eV)
TZ-1	5.01	3	2.01
TZ-2	5.21	3	2.21

Table 2. The energy band gap of TZ-1 and TZ-2 obtained from UV results and DFT calculations.

Molecular	Eg (eV)	Eg (DFT, eV)
TZ-1	2.01	2.39
TZ-2	2.21	2.52
